# Planktonic Rotifers in a Subtropical Shallow Lake: Succession, Relationship to Environmental Factors, and Use as Bioindicators

**DOI:** 10.1155/2013/702942

**Published:** 2013-06-20

**Authors:** Gaohua Ji, Xianyun Wang, Liqing Wang

**Affiliations:** ^1^College of Fisheries and Life Science, Shanghai Ocean University, Shanghai 201306, China; ^2^College of Environmental Science and Engineering, Tongji University, Shanghai 200092, China; ^3^Shanghai National Engineering Research Center of Urban Water Resources Co., Ltd., Shanghai 200082, China

## Abstract

Changes in the density and species composition of planktonic rotifers as well as their relationship to several environmental variables were studied at Dadian Lake, a shallow subtropical lake, which was completely dredged and reconstructed. Samples were taken monthly (2006–2009) at five stations. The total rotifer abundance exponentially declined and reached a relatively stable stage in 2009. *Polyarthra dolichoptera* and *Trichocerca pusilla* dominated the rotifer community in most seasons. TN, TP, and COD_Mn_ went down at the beginning of the monitoring period, rebounded in the second winter, and then decreased and reached a stable state in 2009. CCA showed that the most significant variations were caused by fluctuations in temperature, COD_Mn_, SRP, and NO_2_-N. The rotifer community experienced a two-stage succession and the difference of species between the stages was exhibited during warm seasons. GAMs indicated that the selected factors were responsible for 64.8% of the total rotifer abundance variance and 16.5*~*64.3% of the variances of individual species abundance. Most of the environmental parameters had effects on rotifer abundance that could only be described by complicated curves, characterised by unimodality and bimodality instead of linearity. Our study highlighted the temperature influence on rotifer species composition and total abundance in subtropical lakes.

## 1. Introduction

In aquatic ecosystems, among the earliest responses to stress are changes in the species composition of small, rapidly reproducing species with wide dispersal powers [1]. Zooplankton is composed of organisms with high environmental sensitivity, which can be used as bioindicators of environmental changes [2]. Rotifers are small animals and react faster to changes in water conditions than other zoological groups of freshwater zooplankton due to their short development cycle (*r* strategists). They are considered to be the most sensitive group to physical and chemical environmental changes [3].

Many studies have focused on rotifer response to abiotic factors, and some have tried to establish one-to-one causal relationships between rotifer composition and trophic conditions [4–11]. Most of these studies have either explored relationships between rotifers and environmental factors based on linear models or studied the distribution of species using redundancy analysis (RDA) [12, 13]. However, (generalised) linear models are not the most adequate models for rotifer abundance-environmental factor processes. Castro et al. studies rotifer community structure in three shallow lakes using correspondence analysis (CCA), which assumes a unimodal response model to environmental gradients [4]. The use of CCA in community ordination deserves more attention.

The generalised additive model (GAM) is an extension of the generalised linear model [14]. The advantage of the GAM lies in its adaptability for nonnormally distributing variables. As a flexible and effective technique for dealing with nonlinear relationships between the response and the set of explanatory variables, it is less restrictive in its assumptions concerning the underlying distribution of data. The model assumes that the dependent variable is dependent on the univariate smooth terms of the independent variables rather than the independent variables themselves [15]. The basic GAM model used for this study follows this formula:

(1) g(Y ∣ X1,X2,…,Xp) =α+f1(X1)+f2(X2)+⋯+fp(Xp),

where *g*(·) is a link function, and *f*_*i*_(*X*_*i*_),  *i* = 1,2,…, *p* are nonparametric smooth functions (smoothing spline) for independent variable *X*_*i*_. The function *f*_*i*_ is estimated in a flexible manner and does not have to be nonlinear for all independent variables in the GAM. The model is a useful scientific tool applied in many scientific fields [15–18].

In this study, the physicochemical and biological characteristics of a shallow sediment-dredged lake were investigated over a period of four years. GAMs as well as other statistical analyses were conducted to (a) test the hypotheses that it would take a long time for the rotifer community to reach a relatively stable state after sediment dredging in the lake and that rotifer diversity indices could reflect the trophic states and (b) investigate the way that environmental factors affected the rotifer abundance (including the total and individual species abundance) and species compositions.

## 2. Materials and Methods

### 2.1. Study Area

**Figure 1:**
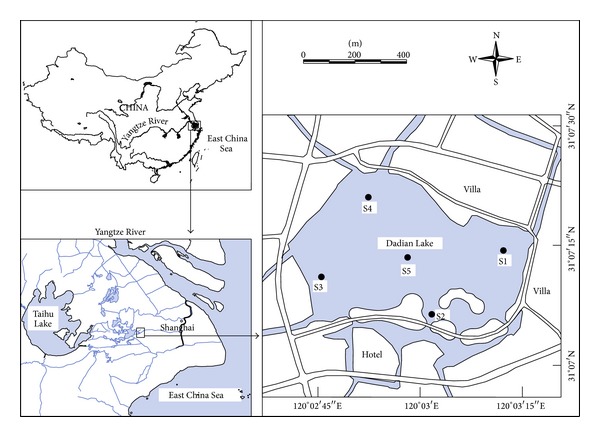
Location of Dadian Lake, China, and the sampling stations.

Dadian Lake (31°07′N, 120°03′E) is located in the lower reaches of the Yangtze River (Figure 1) in China. It is a small, subtropical, shallow, and freshwater lake with an area of 50 ha. The average and maximum depths are 3.0 m and 4.0 m, respectively. The lake surface is 2.56 m above sea level. The hydraulic residence time of the lake is more than 0.5 years. There are hotels, restaurants, and villas nearby, and there are typically yachts on the lake. The lake was previously used for aquaculture; thus, before 2005, cyanobacterial blooms occurred frequently during the summer due to nutrient enrichment from fish aquaculture.

The lake was drained completely, insolated and dredged from July 2005 to May 2006. Dredged silt from the lake was heaped to construct islands in the southern lake. The lakeshore was also reconstructed and consolidated. After the comprehensive improvement, the lake was refilled with water from the northwest river, essentially making it a newly constructed lake. Meanwhile, submerged macrophytes were planted in the lake from May to July 2006. The macrophytes were limited to the littoral zone during our study, which comprised an area of *ca. *15% of the lake. The lake was protected and less affected by anthropogenic activities thereafter.

### 2.2. Sampling

Samples were taken monthly from July 2006 to Dec 2009 at five stations (Figure 1). Depth-integrated samples were obtained from mixed water collected from the bottom to the surface at an interval of 0.5 m using a modified 5-l sampler for water and zooplankton analysis. Chemical parameters, including total nitrogen (TN), nitrite nitrogen (NO_2_-N), nitrate nitrogen (NO_3_-N), total phosphorus (TP), soluble reactive phosphorus (SRP), and chlorophyll a (chl.a), were measured for each sample using standard methods [19]. Ammonium-nitrogen (NH_4_-N) and the potassium permanganate index (COD_Mn_) were determined using the nesslerisation and potassium permanganate index methods, respectively [20]. Temperature (Tem) was determined using a mercury thermometer.

For qualitative analyses, rotifer samples were collected by vertical hauls using a 50 *μ*m mesh net with a reducing cone. Species were identified according to [21, 22]. For quantitative analyses, the sedimentation method was used to concentrate 1 litre of a depth-integrated sample. Counting was performed under a microscope in a 1 mL Sedgwick-Rafter chamber [23].

Crustacean samples were collected by vertical hauls for qualitative analyses and by filtering 20 litres of depth-integrated water through a 50 *μ*m mesh net for quantitative analyses [13]. The entire sample was counted under a microscope. Juvenile (nauplii and copepodite) copepods were also counted, but only the copepodite and adult were included in the total copepod density. Crustaceans were recorded from July 2006 to Oct 2007 and from Jan 2009 to Dec 2009.

### 2.3. Data Analysis

The Shannon-Wiener index (*H*′), Pielou's evenness index (*J*), and Margalef's index (*D*_Mg_) were calculated using the formulas: *H*′ = −*P*_*i*_Σ log⁡_2_ *P*_*i*_,   *J* = *H*′/log⁡_2_ *S*,  and  *D*_Mg_ = (*S* − 1)/ln⁡*N*, where *P*_*i*_ is the proportion of individuals belonging to the *i*th species, *S* is the number of species in the sample, and *N* is the density of all species in the sample [12, 24].

Notched box plots were used to detect the differences between the seasonal rotifer densities. If the notches for two medians do not overlap in the display, the medians are approximately significantly different at about a 95% confidence level [25]. The notched box plots were provided by Origin 8.5 (OriginLab Corporation, Northampton, MA, USA).

CCA was used to reduce the data dimensionality and identify the main variables affecting rotifer community structure. We opted for a unimodal model of ordination instead of a linear one because the GAMs showed the nonlinear effects of environmental factors on rotifers. Even though the preliminary DCA (detrended correspondence analysis) showed a short gradient length on the biological data (SD = 1.96), we were still able to use CCA [26]. To reduce the influence of spatial variability, the species and environmental data were averaged for each sampling occasion. This resulted in 35 samples, with 26 main species (comprising >5% in more than three samples before averaging). For this analysis, we used the rotifer species and the values of abiotic variables and chlorophyll a (log-transformed data). The statistical significance of the first and all the ordination axes was tested using a Monte Carlo permutation test (4999 unrestricted permutations). CCA and DCA were conducted using CANOCO for windows 4.5 (Biometris-Plant Research International, Wageningen, The Netherlands).

GAM was used to assess the effects of the environmental factors on rotifer densities. A preliminary step-wise GAM was performed to determine the best-fitting model. The response variable in the model was the log⁡(*x* + 1)-transformed rotifer density (total or individual species presented in more than 18 samples), and the explanatory variables were abiotic factors and chl.a. GAM analysis and plots were performed using S-PLUS 8.0 (Insightful Corporation, Seattle, WA, USA). Considering that crustacean (copepod and cladoceran) records were not complete and that they presented themselves in extremely low densities in cold seasons, they were not included in GAM analysis. Pearson partial correlation analysis was performed between rotifer, cladoceran, and copepod, using the PROC CORR procedure of SAS 9.1 (SAS Institute Incorporated, Cary, NC, USA).

## 3. Results

### 3.1. Abiotic Parameters

**Figure 2:**
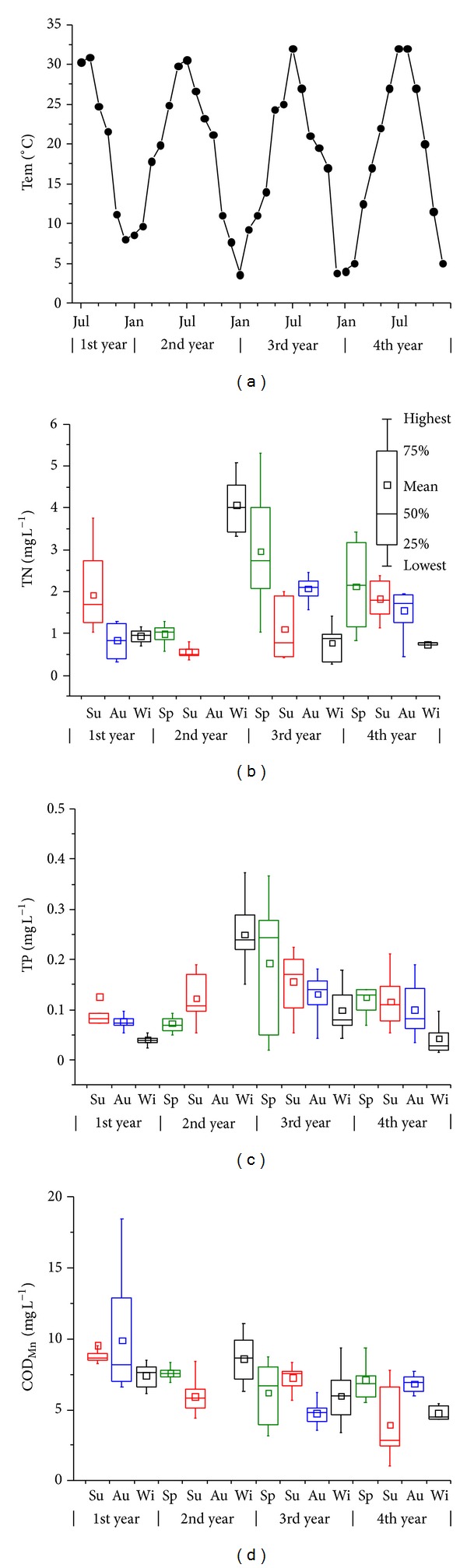
Seasonal dynamics of Tem, TN, TP, and COD_Mn_ in Dadian Lake from 2006 to 2009. Outliers (>*Q*3 + 1.5Δ*Q*  or <*Q*1 − 1.5Δ*Q*,  Δ*Q* = *Q*3 − *Q*1) are not shown in the plots. Sp: spring (from Mar to May), Su: summer (from June to Aug), Au: autumn (from Sep to Nov), Wi: winter (from Dem to Feb). No data were recorded in the 2nd autumn except for Tem.

All of the abiotic parameters fluctuated strongly during the study period (Figure 2). The highest water temperature was 32°C and the lowest was 3.6°C. TN and TP were similar in the seasonal dynamics (*R* = 0.55, *P* < 0.0001). They decreased shortly after lake refilling and increased in the next year, with the highest value occurring during the 2nd winter (TN: 4.06 mg L^−1^, TP: 0.25 mg L^−1^, on average). In the 3rd and 4th years, there was a declining trend from spring to winter. The lowest TN values were 0.60 (2nd winter) and 0.74 mg L^−1^ (4th winter), and the lowest TP value was 0.043 mg L^−1^ (4th winter). COD_Mn_ also rebounded during the 2nd winter and it declined overall, without any distinctive pattern.

The monthly average and maximum and minimum values for NH_4_-N, NO_2_-N, NO_3_-N, and SRP were 0.58 (0.018–2.18), 0.049 (0.0023–0.206), 0.28 (0.012–0.892), and 0.021 (0.0035-0.083) mg L^−1^, respectively. NH_4_-N and SRP peaked in the 2nd winter, and NO_2_-N peaked in the 3rd and 4th winters.

### 3.2. Rotifers

A total of 91 rotifer species belonging to 18 families and 29 genera were recorded during the study period, including 26 abundant species (comprising >5% in more than three samples) (Table 1). The total species numbers of *Brachionus, Keratella,* and *Trichocerca* accounted for nearly half of the abundant species. Species richness was relatively low in winter.

**Figure 3:**
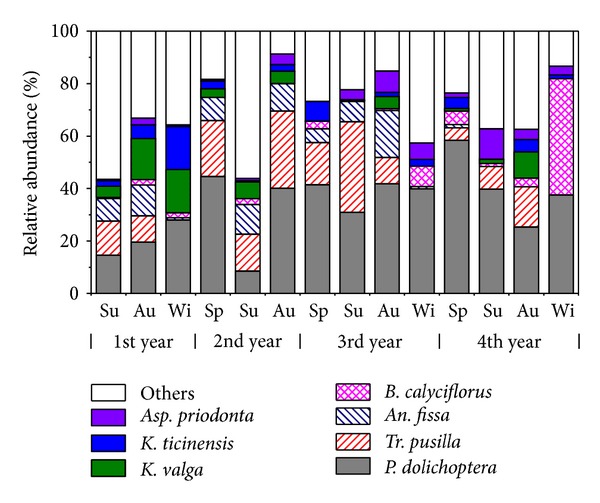
Seasonal variations of the relative abundance of the most representative rotifer species (average relative abundance >3%) from 2006 to 2009 in Dadian Lake (no data recorded during the 2nd winter).

The rotifer community varied with the season. *Polyarthra dolichoptera* was classified as superdominant (seasonal relative abundance >30%), while *Tr. pusilla *was eudominant (>10%) in most seasons (Figure 3). They accounted for 46.3% of the total rotifer abundance on average. *Tr. pusilla* and *B. calyciflorus* were superdominant in the 3rd summer and in the 4th winter, respectively. *B. forficula*, *Cephalodella inquila*, *K. valga*, *K. ticinensis*, *Lecane elachis,* and *Tr. Longiseta* were eudominant in the seasons of the 1st and 2nd years, and *Asplanchna priodonta*, *Hexarthra mira*, *K. quadrata*, *K. cochlearis,* and* Dicranophorus forcipatus* were eudominant in the seasons of the 3rd and 4th years. *Anuraeopsis fissa* was eudominant in autumn, except for autumn of the 4th year. It is noticeable that *An. fissa*, *B. forficula, *and* L. elachis *were eudominant but *P. dolichoptera* was not eudominant in the 2nd summer.

**Figure 4:**
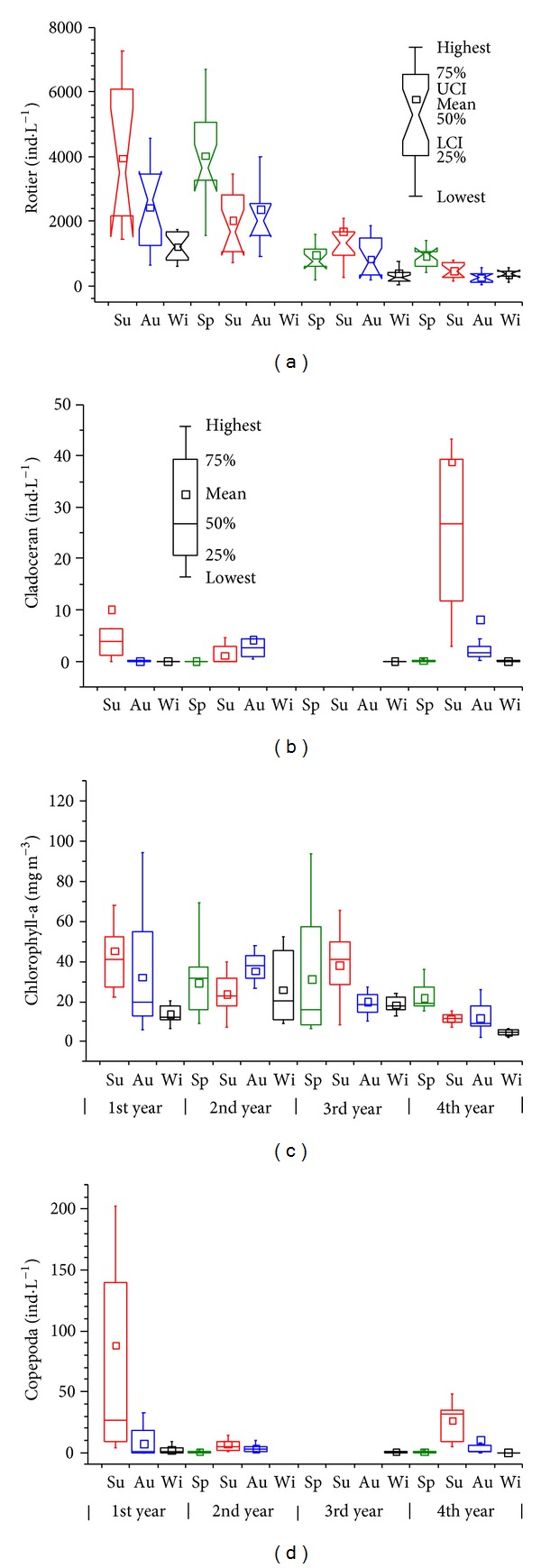
Seasonal variations of the total rotifer density and biotic parameters (chlorophyll a, cladoceran, and copepoda). LCI, UCI: lower and upper bound of 95% confidence interval of median, respectively. (If the notches of two box plots do not overlap, we can assume that the two median values differ at a 95% confidence level.) No data were recorded for rotifers in the 2nd winter or for crustaceans in the 3rd year.

The total rotifer density exhibited a clear decrease from 2006 to 2009. The equation describing this is ln (rotifer density) = 8.39 − 0.171 t (*R* = 0.8789, *P* < 0.001) (Figure 4). The density was very high and fluctuated greatly in the 1st and 2nd years but decreased in the 3rd and 4th years and became more stable. The rotifer densities in each season of the 4th year were lower than those in the 1st or 2nd year (*α* = 0.05). The highest density occurred in spring or summer, and the lowest occurred in winter within one year.

### 3.3. Biotic Parameters

Chlorophyll a showed a similar pattern to TN and TP; that is, it decreased in the 1st year, then increased, and decreased again during the 3rd and 4th years. However, Pearson correlation analysis revealed that there was no significant relationship between chl.a and TN or TP. The highest seasonal chl.a was 45.3 mg m^−3^ (1st summer) and the lowest was 4.5 mg m^−3^ (4th winter). 

Cladoceran and copepod densities were found to be extremely low, except in the summer and autumn during the study period. The main cladoceran species were *Diaphanosoma dubium* and *Bosmina* spp. (*B. longirostris* and *B. coregoni*), both in 2006-2007 and 2009, while *Bosminopsis deitersi* and *Moina* spp. (*M. micrura, Moina brachiata*) appeared in large numbers in 2009. The copepods mainly consisted of Cyclopoida (*Thermocyclops* spp.), which is a carnivorous species, both in 2006-2007 and 2009. Cladoceran peaked in the 4th summer (39 ind. L^−1^), while the highest copepod density occurred shortly after the lake was refilled (1st summer, 88 ind. L^−1^). Cladoceran density was negatively correlated with the rotifer population (*R* = − 0.223,  *P* = 0.0083, *n* = 140), while copepod density was positively associated with it (*R* = 0.235, *P* = 0.0053, *n* = 140).

### 3.4. Statistical Analysis

**Figure 5:**
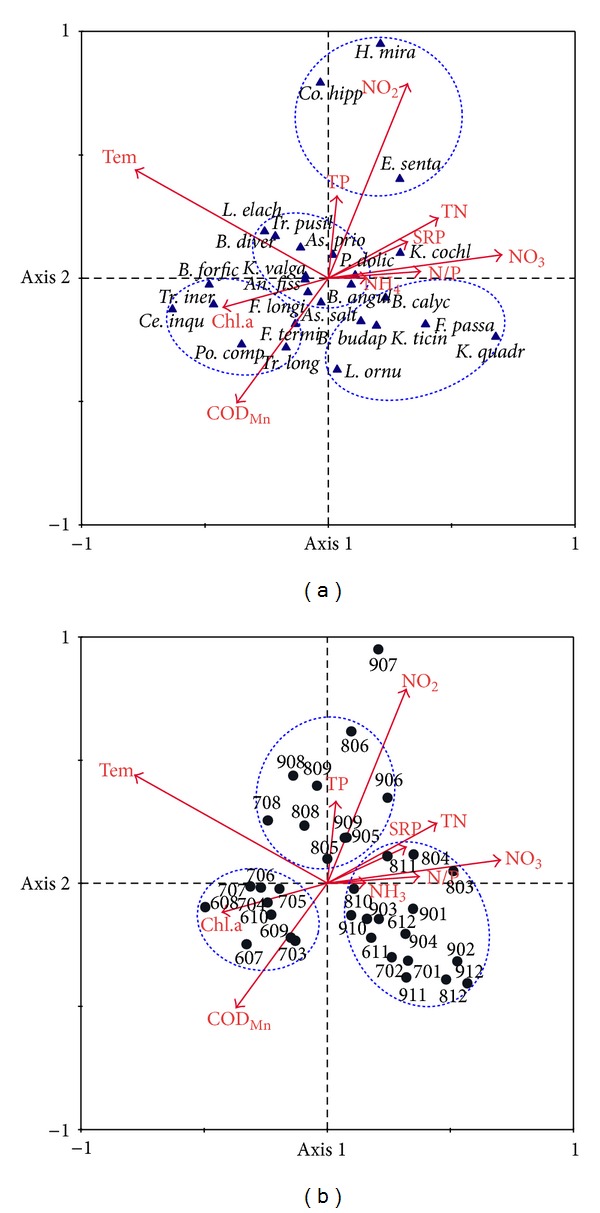
CCA ordination plots. Species abbreviations are presented in Table 1. (a) Species and environmental variables. (b) Samples and environmental variables. Numbers represented sample times, for example, 907 means Jul 2009. The eigenvalues for the first two axes are 0.18 and 0.126. The Monte Carlo permutation test was significant on the first axis (*F* = 3.965, *P* = 0.002) and on all axes (*F* = 1.885, *P* = 0.002).

The first two ordination axes explained 24.1% of the variance of the species data in CCA (Figure 5). Forward selection and associated Monte Carlo permutation tests of the significance of the environmental variables (Table 2) indicated that Tem, NO_2_-N, SRP, COD_Mn_, and NO_3_-N accounted for most of the variation in species distribution when considered by themselves (*λ*-1). After the addition of Tem to the ordination, only NO_2_-N accounted for any significant amount of the remaining variation (*P* < 0.05) (*λ*-A). The ten variables in Table 2 explained 55.9% of the total variance in the species data. Tem, chl.a, and COD_Mn_ were negatively associated with axis 1, but NO_3_-N and TN were positively associated with axis 1. NO_2_-N, TP, and Tem were positively associated with axis 2, and COD_Mn_ was negatively associated with axis 2.

The CCA revealed four main groups of species. Some of the abundant species, for example, *Tr. inermis*, *B. forficula,* and *Ce. inquilina, *preferred high Tem and COD_Mn_ (Figure 5(a), bottom-left quadrant), and they mostly presented themselves in warm seasons during the first two years (Figure 5(b), bottom-left quadrant). *H. mira*, *E. senta,* and *Co. hippocrepis *preferred high NO_2_-N and low COD_Mn_ and were abundant in warm seasons of the last two years. *K. quadrata*, *F. passa,* and* B. calyciflorus* populations grew in cold season. *As. priodonta* and other species preferred warm temperature and occurred in every year of the study.

The CCA also showed that the rotifer community experienced a two-stage succession (Figure 5(b)). The difference between the stages was exhibited during warm seasons. In the first stage, the rotifer community was mainly composed of *Po. complanata*, *Ce. inquilina,* and other species. In the second stage, *H. mira, Co. hippocrepis, E. senta,* and other species dominated the community. There were also some species that occurred throughout the study period, such as *P. dolichoptera* and *K. valga*. In the cold seasons, the species in the rotifer community were similar in different stages.

**Figure 6:**
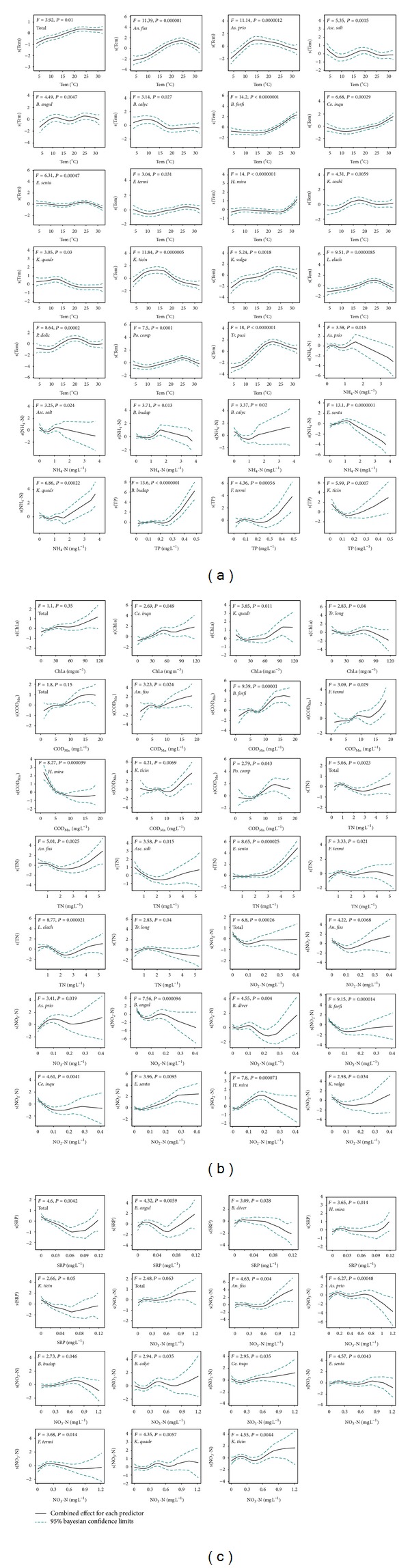
Plots showing the effect of selected environmental parameters on rotifer densities. For the total rotifer density, all of the selected parameters are shown. For the individual species densities, only the selected parameters with significance (*P* < 0.05) are shown: (*n* = 169).

The best general additive models (GAMs) determined using a stepwise procedure, are shown in Table 3. The environmental factors selected by the procedure differed greatly with respect to different species and can explain 16.5~64.8% of the response variance. Environmental parameters included in the GAM for total rotifer density explained approximately 64.8% of the variations in the total rotifer density. NO_2_-N, TN, SRP, and Tem were significant while chl.a, NO_3_-N, and COD_Mn_ were not significant at a 5% level. The effects of the selected environmental parameters on rotifer densities are shown in Figure 6. 

Total rotifer density increased with the increase in temperature, reaching its maximum at approximately 23°C but decreased slightly when the temperature exceeded 25°C. Eighteen species out of the 22 frequent species (present in >18 samples) were significantly affected by temperature. There are four main types of rotifer responses to temperature. One type preferred high temperature (25–30°C), including *B. forficula*, *Ce. inquilina,* and *H. mira*. The second type, consisting of most of the abundant species, that is, *An. fissa*, *E. senta*, *F. terminalis*,* K. valga*, *L. elachis*, *P. dolichoptera*, *Po. complanata*, and *T. pusilla, *peaked at a temperature of 20–25°C. Therefore, the total rotifer density was largest at 23°C. The third type peaked at approximately 15°C, and this type includes *As. priodonta*, *K. cochlearis,* and *K. ticinensis*. The fourth type preferred a low temperature (*K. quadrata* and *B. calyciflorus*). Interestingly, *B. angularis *had two peaks at approximately 12 and 25°C.

Chl.a was only significant in the GAMs of three species (*Ce. inquilina*, *K. quadrata,* and *T. longiseta*). The total rotifer density showed an overall increase with increasing chl.a but decreased during 30–50 mg m^−3^, although not significant. The confidence intervals broadened when chl.a was greater than 40 mg m^−3^ because few samples had high numbers of chl.a. Six species responded significantly to COD_Mn_, and their fitting cures greatly varied. *H. mira* decreased with the increase of COD_Mn_.

Only three GAMs identified TP as an explanatory variable, and seven GAMs identified TN as an explanatory variable. *B. budapestinensis* and *F. terminalis* had a similar curve fit for TP with a peak before 0.2 mg L^−1^, but the curve for *K. ticinensis *was at a minimum there. *E. senta *increased with increased TN, but the others did not exhibit a trend.

Inorganic ions also had some effects on rotifer abundance. *E. senta* increased with NH_4_-N when it was lower than 1 mg L^−1^. The fitting-curve confidence interval broadened when NH_4_-N was greater than 1.5 mg L^−1^ because there were a few data in that range. Most of the curves for NO_2_-N and NO_3_-N were wavy, but *E. senta *showed a clear trend with increasing NO_2_-N. There was a negative effect of SRP on the total density and the densities of the three species in the range from 0.02 to 0.06 mg L^−1^.

### 3.5. Rotifer Community as Bioindicators

**Figure 7:**
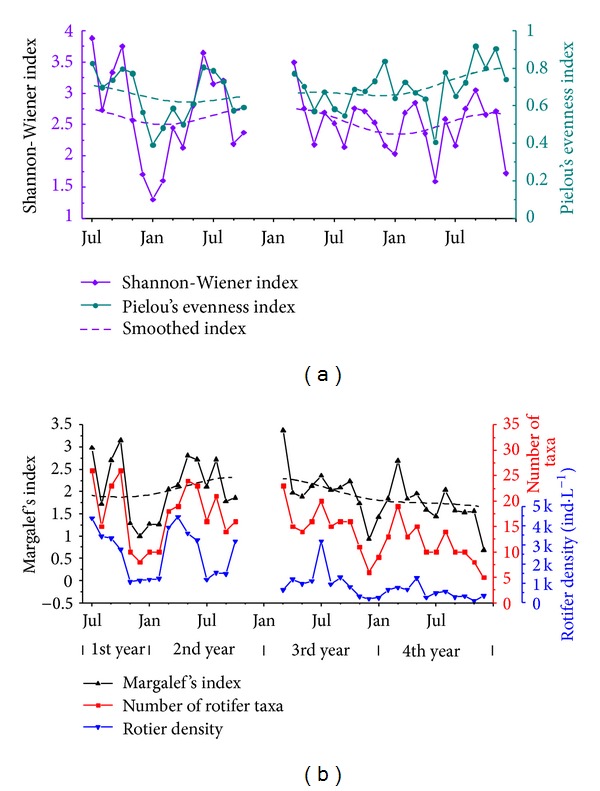
Rotifer diversity indices in Dadian Lake. Smoothed method: FFT filter, six points.

All of the diversity indices varied monthly, but they exhibited little annual fluctuation (Figure 7). *H*′, *J*, and species richness (*R*) were strongly positively correlated with Tem. *D*_Mg_ was positively associated with chl.a and TP. *H*′ and *R* were positively associated with chl.a. There was a positive correlation between *R*, *H*′, and *D*_Mg_, but only *J* is related to *H*′(Table 4). The average values of *H*′, *J*, and *D*_Mg_ were 1.95, 0.68, and 2.56, respectively. 

There was a positive correlation between *D*_Mg_ and *N*. When temperature rose, both *R* and *N* increased, but *R* increased more than ln⁡*N*, so *D*_Mg_ increased. Both *D*_Mg_ and *N* were positively correlated with Tem, resulting in a positive correlation between *D*_Mg_ and *N*.

## 4. Discussion

Sediment dredging disrupted the ecological balance and destroyed the benthos and aquatic vegetation [27, 28]. Dredging likely took the zooplankton resting eggs away, but the sediment was used to create islands in the southern area of the lake. These islands have become important banks for rotifer resting eggs. We only have a few data regarding the zooplankton before dredging. The average rotifer density was 3573 ind. L^−1^ in April 2005. However, the intensive dredging and construction rendered the lake analogous to a newly constructed one, and we cared more about the succession of rotifer community after the lake was refilled. The rotifer community mainly originated from the river and from the resting egg banks. The refilled water from the connected rivers was low quality, with a TN of 5.05 mg L^−1^ and a TP of 0.34 mg L^−1^, both of which decreased shortly after refilling. The absence of a relationship between TN, TP, and Chl-a may be partly explained by the macrophytes. Because macrophytes compete with phytoplankton, nutrient and chlorophyll a will decrease as the macrophyte population increases [29]. Furthermore, fish and other zooplankton can also constrain TP : Chl-a ratios. Unfortunately, we do have not any exact biomass for them. 

There was a clear change in the rotifer community, both in density and species structure, after refilling. The rotifer density exponentially declined over the four years, but TN and TP rebounded in the 2nd winter and spring. They were all shown low values in the 4th year with smaller variations. This may indicate that the ecosystem in the water column reached a stable state. This phenomenon has also been observed in newly constructed reservoirs [30]. In addition to the density variations, there were also changes in the dominant species of the rotifer community. *P. dolichoptera* and *Tr. pusilla* were widely found and dominated most subtropical lakes, from mesotrophic to eutrophic [13, 23, 31]. They also dominated Dadian Lake during most of the study period, but other dominant species changed; for example, there were more *An. fissa* in the first two years and more *H. mira *in the later years, as mentioned above.

The rotifer community variations were correlated with changes in abiotic and biotic environmental factors. Abiotic factors affected the rotifers directly or indirectly. Temperature and some toxic ions may have directly affected the rotifers, and temperature accounted for a relatively high proportion of the variability in the rotifer community. The total rotifer density reached its maximum at approximately 23°C, but individual species differed in their temperature preferences in this study.

Rotifers generally have a very wide tolerance to temperature, but in separate lakes they are often strongly restricted by and connected with temperature differences [32]. *P. dolichoptera* peaked at approximately 20°C and was a perennial superdominant in the present study, but it was considered to be a “winter species” by [24, 33]. However, other studies also found that it could occur at higher temperatures in small lakes and ponds [32]. *As. priodonta* and *F. terminalis* were considered to prefer temperatures below 10°C [24], but they peaked at 15 and 25°C in our study and behaved similarly in another study [32]. *B. calyciflorus* was found to prefer low temperatures in this study but not in others [32]. There is some agreement among different studies; for example, *K. quadrata* preferred low temperatures and *T. pusilla* peaked at high temperatures in our study as well as in other studies [24, 31, 34]. The thermal preference discrepancy of the same species between different individual lakes may be attributed to the fact that temperature alone does not generally decide when and where a species occurs. Other abiotic and biotic factors also play a role [32].

Some abiotic factors are toxic to rotifers. Chen et al. [35] observed that a nitrite concentration of 10 mg L^−1^ NO_2_-N markedly inhibited the growth of *B. calyciflorus*. Although the tolerance of *B. calyciflorus* to nitrite was high, lower nitrite levels may have increased the production of microcystin. Furthermore, nitrite and microcystin could have acted in a synergistic manner, causing toxicity. Schlüter and Groeneweg [36] found that the reproduction of *B. rubens* was unaffected up to a concentration of 3 mg L^−1^ of ammonia, and, in the range of 3–5 mg L^−1^, the reproduction rate decreased, but none died, and there was a trend of declining populations for most species with NH_4_-N > 2 mg L^−1^ in our study. However, in our study, the nitrite and NH_4_-N concentrations were found to be below 0.5 and 4 mg L^−1^, respectively. The effects of nitrate and ammonia on some species in this study are more likely to be indirect, and further investigation is needed.

Abiotic factors, including NH_4_-N, NO_3_-N, NO_2_-N SRP, TN, and TP, can indirectly affect rotifers via trophic cascade. The abundance of these materials has an influence on the quantity and quality of plankton as well as bacteria. Rotifers feed on bacteria; protozoa, including ciliates and heterotrophic flagellates; and algae, including pico- and nanophytoplankton. Many planktonic rotifers are known as relatively unselective microfiltrators, feeding on particles in the range of 0.5 to 20 *μ*m, and other grasping species feed preferentially on larger organisms (esp. ciliates) [37]. Chl.a may be representative of algal quantity and TP was found to positively correlate with chl.a. When chl.a was included in CCA or GAMs, TP could not explain the more significant variations of the rotifer community. TN may influence the plankton community. The average TN : TP of Dadian Lake was 17 : 1. A total N : P ratio below 29 : 1 may indicate the dominance of cyanobacteria in this lake [38]. Limitation of N may favour nitrogen-fixing cyanobacteria. But the dominate phytoplankton species in Dadian Lake were *Phormidium *spp. and *Spirulina *sp., which cannot fix nitrogen. Thus, the increased TN may favour the growth of those cyanobacteria, which cannot be ingested by many rotifers [39]. Moreover, some cyanobacteria species may release toxicants such as microcystin. As a result, the total rotifer density declined with increased TN in the GAM. When TN was greater than 3, there was no limitation of nitrogen to other algae such as green algae, and the total rotifer density increased. The differences in tolerance of rotifer species to TN may reflect their adaption to different algae.

COD is an indicator of the organic matter in water. Organic matter mainly consists of living and dead plants and animal organisms [40]. Algae, bacteria, protozoa, and debris can contribute to COD, and they are considered food for rotifers. Bacteria and protozoa may constitute 20–50% of rotifer food [37]. Therefore, COD can explain more variations in rotifers than chl.a explained in the CCA and the GAM in our study. 

Biotic factors affect rotifers directly. The edible algae density can determine rotifer abundance. Generally, there is a positive correlation between rotifer abundance and chl.a. In this study, a linear relationship between chl.a and total rotifer abundance was observed in the case of chl.a <30 mg m^−3^. However, the rotifer abundance slightly declined with the increase of chl.a when it was greater than 30 and smaller than 60 mg m^−3^, most likely because cyanobacteria, especially *Microcystis,* dominated in this interval. Each suspension-feeding rotifer in a community may have a different food preference; hence, the impact on the ecosystem will be different according to its feeding habits [41]. *Polyarthra *prefers food in a large size range (approximately 1–40 *μ*m) [37], which enables it to dominate throughout the year.

Cladocerans had a negative correlation with rotifer densities in this study. Planktonic rotifers often are abundant when only small cladocerans occur but typically are rare when large cladocerans are present. Cladocerans share available food with rotifers. The main species of cladocerans are filter feeders [4–11, 42]. They feed on nanoplankton and other small particles, but cladocerans often show dominance over rotifers due to their large body sizes and other factors [43]. The extremely low density of cladocerans in this lake could be attributed to fish predation. There were approximately 11000 kg of fish, mainly composed of silver and bighead carp, released to the lake after it was refilled. Though the primary purpose of fish release was to inhibit the potential *Microcystis* bloom by direct predation, the fish also predated the large zooplankton population, especially cladocerans, because of their nonselective filtering habit. The rotifers may benefit from this fish behaviour. The observed copepods mainly consisted of Cyclopoida, which prey on rotifers [44, 45]; however, there was no significant relationship between them detected in our study, most likely due to the low prey pressure on rotifers.

 It has been often observed that the abundance of rotifers is proportional to the trophic status of a water body [23]. Many rotifer indices were established to evaluate the lake trophic status. The average values of *H*′, *J*, and *D*_Mg_ indicate that the lake was somewhat mesotrophic. However, these indices exhibited poor relationships to TP, TN, and chl.a. Compared to the nutrient concentration, the relatively high index was most likely due to the instability of the lake ecosystem after dredging. According to the intermediate disturbance hypothesis, disturbance should promote biodiversity. Furthermore, a diversity of aquatic environments, such as islands and macrophytes, may provide more niches.

More complex indices for rotifers, including the saprobic index and *Q*_*B*/*T*_ [5], were established for saprobic and trophic evaluation according to rotifer trophic preference. Some studies have established a linear regression formula describing the relationship between trophic status and the rotifer community index [11, 46]. These indices were reliable in the lakes they studied.

However, it is very hard to establish one-to-one causal relationships between rotifer composition and trophic conditions. The responses of rotifers to environmental factors were found to be nonlinear, sometimes unimodal or bimodal, in our study. Nutrient elements indirectly affect rotifers via the food chain. Moreover, apart from trophic conditions, other abiotic factors [47] as well as food composition (esp. algae species), vegetation cover [48], and predation [3] also play important roles in determining the abundance and species composition of the rotifer community. As a result, the trophic preferences of individual rotifer species may differ by region. *Tr. pusilla* occurs in mesotrophic lakes, and *P. dolichoptera* is associated with a low trophic state [5, 6, 49], but they were observed to dominate regardless of changes in trophic status in our study and other studies [31]. Nevertheless, rotifers still have their value when assessing trophic conditions. Some authors recommended using rotifer abundance to obtain a rough estimate of lake trophic status: 500–1000 ind. L^−1^: mesotrophic or mesoeutrophic, 1000–2500 ind. L^−1^: eutrophic and 3000–4000 ind. L^−1^: moderately eutrophic [9, 31]. Our results fit within those criteria.

## 5. Conclusions 

It would take a long time to completely restore the aquatic ecosystem in a completely dredged lake, because both the total rotifer abundance and abiotic parameters reached a relatively stable stage in the fourth year. CCA showed that changes of rotifer species composition along with trophic state are exhibited in warm seasons. The GAMs indicated that most of the environmental parameters had complex effects on the rotifer abundance, as demonstrated by the complicated curves describing their relationships. Although rotifer species distribution and total abundance can be used to roughly assess trophic state, it is very hard to establish one-to-one causal relationships between the rotifer community and trophic conditions due to the unimodal effects of nutrient elements on rotifers. In addition, temperature had a predominant influence on rotifers compared to that of trophic conditions in subtropical lakes. Rotifers used for bioindicators should be sampled in warm seasons.

## Figures and Tables

**Table 1:** Abundant species in Dadian Lake from 2006 to 2009 (in abundance order).

Rotifer species	Abbreviations
*Polyarthra dolichoptera* Idelson, 1925	*P. dolic *
*Trichocerca pusilla *(Jennings, 1903)	*Tr. pusi *
*Anuraeopsis fissa* Gosse, 1851	*An. fssa *
*Keratella valga* (Ehrenberg, 1834)	*K. valga *
*Brachionus angularis* Gosse, 1851	*B. angul *
*Asplanchna priodonta* Gosse, 1850	*As. prio *
*K. ticinensis* (Callerio, 1921)	*K. ticin *
*B. calyciflorus *Pallas, 1766	*B. calyc *
*Filinia longiseta* (Ehrenberg, 1834)	*F. longi *
*K. cochlearis* (Gosse, 1851)	*K. cochl *
*B. forficula *Wierzejski, 1891	*B. forfi *
*Cephalodella inquilina* Myers, 1924	*Ce. inqu *
*K. quadrata* (Müller, 1786)	*K. quadr *
*Ascomorpha saltans* Bartsch, 1870	*Asc. salt *
*B. diversicornis* (Daday, 1883)	*B. diver *
*Hexarthra mira* (Hudson, 1871)	*H. mira *
*Lecane elachis* (Harring & Myers, 1926)	*L. elach *
*T. longiseta* (Schrank, 1802)	*Tr. long *
*Epiphanes senta* (Müller, 1773)	*E. senta *
*B. budapestinensis* Daday, 1885	*B. budap *
*Conochilus hippocrepis* (Schrank, 1803)	*Co. hipp *
*F. passa* (Müller, 1786)	*F. passa *
*F. terminalis* (Plate, 1886)	*F. termi *
*L. cornuta* (Müller, 1786)	*L. cornu *
*Pompholyx complanata* Gosse, 1851	*Po. comp *
*T. inermis* (Linder, 1904)	*Tr. iner *

**Table 2:** Results of forward selection and Monte Carlo permutation tests from CCA of rotifer species. Environmental variables are listed by the order of their inclusion in the model (*λ*-A).

Factors	*λ*-1	*λ*-A	*P*(*λ*-1)	*P*(*λ*-A)
Tem	0.145	0.145	0.0002	0.0002
NO_2_-N	0.104	0.107	0.0006	0.0002
SRP	0.057	0.047	0.068	0.075
COD_Mn_	0.075	0.046	0.008	0.084
NO_3_-N	0.108	0.045	0.0006	0.089
TP	0.043	0.038	0.29	0.20
Chla	0.053	0.03	0.112	0.46
N/P	0.041	0.037	0.36	0.23
NH_4_-N	0.034	0.029	0.55	0.47
TN	0.062	0.035	0.05	0.28

**Table 3:** GAMs generated by the forward and backward stepwise selection procedure.

Response variables	Explanatory variables selected by stepwise procedure	Explained variance (%)
Total	s(chl.a) + s(NO_2_-N) + s(NO_3_-N) + s(TN) + s(SRP) + s(COD_Mn_) + s(Tem)*	64.8
*An. fissa *	s(chl.a) + s(NO_2_-N) + s(NO_3_-N) + s(TN) + s(COD_Mn_) + s(Tem)	57.6
*Asc. Saltans *	s(NH_4_-N) + s(TN) + s(Tem)	20.2
*Asp. priodonta *	s(NH_4_-N) + s(NO_2_-N) + s(NO_3_-N) + s(Tem)	34.3
*B. angularis *	s(NO_2_-N) + s(NO_3_-N) + s(SRP) + s(Tem)	30.7
*B. budapestinensis *	s(NH_4_-N) + s(NO_3_-N) + s(TP)	35.7
*B. calyciflorus *	s(NH_4_-N) + s(NO_3_-N) + s(Tem)	20.5
*B. diversicornis *	s(NO_2_-N) + s(SRP) + s(COD_Mn_) + s(Tem)	34.4
*B. forficula *	s(chl.a) + s(NO_2_-N) + s(NO_3_-N) + s(COD_Mn_) + s(Tem)	64.3
*Ce. inquilina *	s(chl.a) + s(NH_4_-N) + s(NO_2_-N) + s(NO_3_-N) + s(Tem)	48.6
*E. senta *	s(NH_4_-N) + s(NO_2_-N) + s(NO_3_-N) + s(TN) + s(Tem)	41.1
*F. longiseta *	s(COD_Mn_) + s(Tem)	36.4
*F. terminalis *	s(NO_3_-N) + s(TN) + s(TP) + s(COD_Mn_) + s(Tem)	34.0
*H. mira *	s(NO_2_-N) + s(SRP) + s(COD_Mn_) + s(Tem)	64.3
*K. cochlearis *	s(NO_3_-N) + s(Tem)	17.1
*K. quadrata *	s(chl.a) + s(NH_4_-N) + s(NO_3_-N) + s(Tem)	36.9
*K. ticinensis *	s(NO_3_-N) + s(SRP) + s(TP) + s(COD_Mn_) + s(Tem)	53.4
*K. valga *	s(chl.a) + s(NO_2_-N) + s(NO_3_-N) + s(TN) + s(Tem)	44.7
*L. elachis *	s(chl.a) + s(TN) + s(COD_Mn_) + s(Tem)	34.5
*P. dolichoptera *	s(COD_Mn_) + s(Tem)	21.1
*Po. complanata *	s(chl.a) + s(NO_2_-N) + s(COD_Mn_) + s(Tem)	33.2
*Tr. longiseta *	s(chl.a) + s(TN) + s(COD_Mn_)	16.5
*Tr. pusilla *	s(chl.a) + s(NO_3_-N) + s(TN) + s(Tem)	61.1

*The GAM formula is ln(densities + 1) ~ s(chl.a) + s(NO_2_-N) + s(NO_3_-N) + s(TN) + s(SRP) + s(COD_Mn_) + s(Tem).

**Table 4:** Correlations between the diversity indices, including the Shannon-Wiener index (*H*′), species richness (*R*), evenness index (*J*), Margalef's index (*D*_Mg_), total rotifer density (*N*), and environmental factors.

	*H*′	*J*	*D*_Mg_	*R*	*N*	Chl.a	TN	TP	Tem
*H*′	1	0.705	0.768	0.702	0.316	0.344	0.165	0.238	0.435
		**<0.0001**	**<0.0001**	**<0.0001**	0.053	0.037	0.342	0.168	0.006
*J*	0.705	1	0.138	0.019	−0.204	0.054	0.082	0.048	0.079
	**<0.0001**		0.410	0.909	0.219	0.753	0.638	0.783	0.636
*D*_Mg_	**0.768**	0.138	1	0.957	0.505	0.365	0.242	0.380	0.451
	**<0.0001**	0.410		**<0.0001**	0.001	0.027	0.161	0.024	0.005
*R*	0.702	0.019	0.957	1	0.716	0.442	0.093	0.256	0.490
	**<0.0001**	0.909	**<0.0001**		**<0.0001**	0.006	0.594	0.138	0.002
